# A commentary on ‘Comparison of safety and effectiveness between robotic and laparoscopic major hepatectomy: A systematic review and meta-analysis’

**DOI:** 10.1097/JS9.0000000000000820

**Published:** 2023-10-04

**Authors:** Xin Zhao, Tianyang Mao, Fengwei Gao, Hong Wu

**Affiliations:** a Department of Hepato-Pancreato-biliary Surgery, The People’s Hospital of Leshan, Leshan; b Liver Transplantation Center, State Key Laboratory of Biotherapy and Cancer Center, West China Hospital, Sichuan University and Collaborative Innovation Center of Biotherapy, Chengdu, People’s Republic of China

*Dear Editor,*

Laparoscopic surgery and robotic surgery stand as notable methods within minimally invasive surgery. The debate regarding which is superior continues. Several past studies have shed light on this. For instance, in randomized controlled trial (RCT) outcomes related to the radical resection of middle and low rectal cancer and distal gastrectomy, robotic surgery appears to have an edge over laparoscopic surgery^1,2^. However, in abdominal hernia repair, robotic surgery demonstrates similar outcomes to laparoscopic surgery but entails higher medical expenses^3,4^. Regrettably, there exists no high-quality RCT study comparing robotic and laparoscopic hepatectomy in liver surgery, leading to an evidence gap in safety and efficacy comparisons between the two.

We recently reviewed the meta-analysis by Mao *et al*.^5^, which examined the safety and short-term efficacy of robotic versus laparoscopic hepatectomy. The authors assessed 12 studies involving 1657 patients, concluding that robotic major hepatectomy (RMH) and laparoscopic major hepatectomy (LMH) had analogous short-term surgical outcomes and tumor sufficiency. Further, RMH was found superior in metrics like intraoperative blood loss, conversion to laparotomy, incidence of severe complications, and postoperative hospital stay. While the findings offer valuable clinical insights, several areas warrant a deeper exploration.

To begin, aside from the 12 studies reviewed by Mao *et al*., two additional studies^6,7^ fit the inclusion parameters during the author’s retrieval timeframe, meriting consideration in the systematic review. Additionally, there appear to be some inconsistencies in the study’s outcomes. The author alternates between terms such as ‘postoperative hospital stay’ and ‘length of hospital stay’ in the abstract and result analysis, leading to potential confusion. Since some included studies presented incongruent baseline data, a meta-regression analysis elucidating the impact of baseline data on result reliability would be beneficial. The authors’ choice between random and fixed effect models remains unspecified; we recommend the inclusion of a subgroup analysis forest plot in supplemental materials for clarity. Lastly, providing a broader range of outcome indicators in the funnel chart could enhance transparency regarding study publication bias.

In summary, we commend the authors for a comprehensive meta-analysis on minimally invasive extensive hepatectomy. Their findings offer pivotal insights into the safety and efficacy comparison of RMH and LMH, guiding future research direction. Emphasis should be on identifying patients who could benefit most from RMH.

## Ethical approval

Not applicable to this study.

## Sources of funding

This research did not receive any specific grant from funding agencies in the public, commercial, or not-for-profit sectors.

## Author contribution

X.Z.: study design and writing; T.M. and F.G.: critical review; H.W.: study supervision.

## Conflicts of interest disclosure

No conflicting relationship exists for any of the authors.

## Research registration unique identifying number (UIN)

1. Name of the registry: not applicable.
2. Unique identifying number or registration ID: not applicable.
3. Hyperlink to your specific registration (must be publicly accessible and will be checked): not applicable.

## Guarantor

Professor Hong Wu, E-mail: wuhong@scu.edu.cn, Liver Transplantation Center, No. 37, Guoxue Lane, Wuhou District, Chengdu, Sichuan Province, People’s Republic of China.

## Date availability statement

All data generated or analyzed during this study are included in this article. The data are available from the corresponding author upon reasonable request.
